# Digestion-Related Enzyme Inhibition Potential of Selected Mexican Medicinal Plants (*Ludwigia octovalvis* (Jacq.) P.H.Raven, *Cnidoscolus aconitifolius* and *Crotalaria longirostrata*)

**DOI:** 10.3390/foods12193529

**Published:** 2023-09-22

**Authors:** Kimberly Calonico, Julian De La Rosa-Millan

**Affiliations:** Tecnologico de Monterrey, Escuela de Ingenieria y Ciencias, BioFoods Research Lab, Av. Eugenio Garza Sada 2501 Sur, Tecnologico, Monterrey 64849, Nuevo Leon, Mexico; juliandlrm@tec.mx

**Keywords:** enzymatic inhibition, *Ludwigia octovalvis*, *Cnidoscolus aconitifolius*, *Crotalaria longirostrata*, antidiabetic properties, digestive enzymes, α-Amylase, α-Glucosidase, lipase, pepsin, enzyme inhibitors

## Abstract

Medicinal plants offer a valuable source of natural compounds with specific and selective bioactivity. These compounds have been isolated since the mid-nineteenth century and are now commonly used in modern medications. *L. octovalvis* (Jacq.) P.H.Raven, *C. aconitifolius*, and *C. longirostrata* are Mexican medicinal plants consumed regularly, and research has shown that they contain bioactive compounds capable of promoting the inhibition of digestive enzymes. This is noteworthy since enzyme inhibitors are bioactive substances that interact with enzymes, diminishing their activity and thereby contributing to the management of diseases and metabolic disturbances. To investigate the activity of these plants, individual analyses were conducted, assessing their proximal composition, bioactive compounds, and inhibition of α-Amylase, α-Glucosidase, lipase, and pepsin. The results revealed that all three plants exhibited enzymatic inhibition. When comparing the plants, it was determined that *C. aconitifolius* had the lowest concentration required for a 50% inhibition in α-Amylase, α-Glucosidase, and lipase, as indicated by the IC_50_ values. For pepsin, *C. longirostrata* demonstrated the lowest IC_50_ value. By understanding the bioactive compounds present in these plants, we can establish the relationship they have with enzymatic inhibition, which can be utilized for future investigations.

## 1. Introduction

Medicinal plants are a valuable source of natural compounds with precise and selective bioactivity. Since the mid-nineteenth century, many of these have been extracted, isolated, purified, and used in modern medications [1]. The WHO reports that 80% of the world’s population uses medicinal plants to satisfy their health needs, and in Mexico alone, more than 4000 species with medicinal attributes have been identified, representing 15% of the country’s total flora [2]. Medicinal plants can be an excellent source of secondary metabolites, some of which are valuable due to their enzyme-inhibiting properties. In many cases, enzymes serve as the molecular targets in various diseases, as they play a crucial role in regulating metabolic pathways. The relative ease in controlling and modifying them via different strategies makes them attractive targets for drug treatments. One of the interesting features related with enzymes is their inhibition characteristics via different mechanisms, related to the molecular characteristics of plant bioactive compounds that reduce their activity and conversion capacity, which could be useful in treating diseases or correcting metabolic imbalances [1].

Inhibitors targeting the enzymes α-glucosidase and α-Amylase have demonstrated efficacy in reducing postprandial hyperglycemia and are commonly used in the treatment of diabetes [3]; as an example, active compounds such as corilagin (1), repandusinic acid A (2), and mallotinin (3) found in *Phyllanthus urinaria* and *Phyllanthus amarus* have been demonstrated to have inhibited α-glucosidase, particularly through 1, which is a mixed-type mode of inhibition, whereas 2 and 3 competitively inhibit α-glucosidase [4].

Research on phenolic compounds derived from various plants has demonstrated their ability to inhibit activities and pancreatic lipase. These lipase inhibitors have the capacity to reduce the absorption of triglycerides [5]. According to reports, plants such as *Aleurites moluccana* (L.), *Acer mono*, and *Baccharis trimera* Less have demonstrated inhibitory activity against pancreatic lipase [6]. Pepsin, an aspartic protease [7], plays a crucial role in the release of amino acids and short peptides from proteins and peptides, and it is highly expressed in the human gastrointestinal tract, which is why it is essential to maintain some of this activity for nutritional purposes. Certain protease inhibitors derived from plants may hinder the digestive function of dietary proteins by inhibiting trypsin and chymotrypsin activities in the human gastrointestinal system. However, scientific evidence also supports their positive effects, such as anti-inflammatory actions in the gut, and these are believed to be achieved through the inhibition of oxygen free radicals released by impaired cells, as proposed in previous studies in vitro and in vivo [8].

Studies investigating the inhibitory effects on α-glucosidase, α-Amylase, and lipase enzymes are commonly conducted to identify substances suitable for addressing and controlling metabolic disorders. Plant extracts and natural products are screened to identify potential substances with inhibitory activity against these enzymes [3]. The search for new, high-quality, affordable, and easily accessible natural compound enzyme inhibitors is a significant focus for drug discovery and research organizations worldwide [1]. Considering the prevalence of type II diabetes mellitus (TII-DM) as a major health concern in the twenty-first century, allopathic medicines remain the primary option for initial management. However, herbal remedies have gained widespread acceptance as alternative treatments, particularly in countries such as Mexico, where medicinal plants hold deep cultural significance and are highly valued [9].

As per the existing literature, *L. octovalvis* (Jacq.) P.H.Raven and *C. aconitifolius* have historically been employed in Mexico and Central America to mitigate and manage a wide array of conditions linked to metabolic disturbances. Regarding *C. longirostrata*, its selection is attributed to its inclusion in Mexican cuisine; while possessing acknowledged bioactive compounds, it has remained relatively underexplored in the scientific literature. This is significant considering the growing prevalence of metabolic disorders in developing nations, with Mexico being prominently affected by disturbances in lipid and glucose metabolism, particularly diabetes. With over 10% of the Mexican population aged 20 years and older impacted by diabetes, it represents a substantial public health concern [10].

*L. octovalvis* (Jacq.) P.H.Raven, commonly known as “clavillo” [10], is a flowering plant that is widespread in tropical regions across the globe. It has a historical use as a beverage for promoting health and treating conditions such as edema, nephritis, hypotension, and diabetes [11]. *C. aconitifolius* belongs to the shrub family and can reach a height of up to 6 m. It features palmate lobed leaves with milky sap and small flowers arranged in dichotomously branched cymes. Known as “chaya” [12], this plant was cultivated as a leafy green vegetable during the pre-Cambrian era in the Maya region of Guatemala, Belize, and Southeast Mexico. It continues to be valued today for its ease of cultivation, high productivity potential, and rich nutritional content, and is used as a food, medicine, and ornamental plant. *C. longirostrata*, also known as “chipilín”, is native to Central America and Mexico, thriving in diverse environments. The tender shoots, leaves, and young stems of this plant have been utilized since pre-Hispanic times for food preparation, beverages, herbal medicine, and as fodder due to their nutritional composition, which includes carotenoids, vitamin C, iron, calcium, proteins, and various phenolic compounds such as flavonoids, saponins, coumarins, tannins, anthraquinones, anthrones, and alkaloids [13].

The aim of this study was to assess the role of bioactive compounds in *Ludwigia octovalvis*, *Cnidoscolus aconitifolius*, and *Crotalaria longirostrata* against some digestive enzymes related to metabolic diseases. These plants were examined because enzyme inhibitors act as bioactive entities that interact with enzymes. This interaction results in a decrease in the functional activity of enzymes, which can contribute to the management of diseases and metabolic imbalances.

## 2. Materials and Methods

### 2.1. General

For all the methods applied in this research, samples of *C. aconitifolius* and *C. longirostrata* were purchased from a local vendor in Monterrey, Mexico. The samples of *L. octovalvis* (Jacq.) P.H.Raven were germinated and cultivated from *L. octovalvis* seeds donated by Dulce Lourdes Morales-Ferra, who has done previous work on *L. octovalvis* [14]. Every material and method will be described in each section below.

### 2.2. Proximal Analysis

For the proximal analysis, the aerial parts of *L. octovalvis* (Jacq.) P.H.Raven, *C. aconitifolius*, and *C. longirostrata* were used. The plant material was milled in a Wiley knife mill (Thomas Scientific, Swedesboro, NJ, USA). The AACC methods 44-15.02, 08-17.01, and 32-10.01 were utilized for the moisture, ash, and ether extract of the plant flour, which were all calculated [15]. protein content was determined using the AOAC method 960.52 [16].

#### 2.2.1. Dietary Fiber

The Megazyme K-TDFR Kit was used to measure the total dietary fiber (TDF), insoluble dietary fiber (FDI), and soluble dietary fiber (SDF) (Megazyme, Wicklow, Ireland). The kit was also used for the AOAC 991.43 [16] and AACC 32-07.01 methods [15].

#### 2.2.2. Soluble Sugars (Sucrose, Glucose, and Fructose)

Following the procedure developed by Karkacier et al. in 2003 [17], soluble sugars were measured. Briefly, liquid nitrogen was used in a mortar to grind 50 mg of leaf sample. After adding one milliliter of nanopure water, the mixture was centrifuged at 10,000 rpm for 15 min. A 0.45 µm membrane filter (VertiPureTM, Vertical R) was used to filter the supernatant after it had been collected. A Waters HPLC with a MetaCarb 87C column and a guard column was used to analyze 20 microliters of the filtrate. At a flow rate of 0.5 mL per min, deionized water was employed as the mobile phase. Using a Waters 410 differential refractometer detector, online detection was carried out, and Empower 3.7 software was used to analyze the results. Analytical standards for fructose (Fluka, Charlotte, Greenwood Village, NC, USA), glucose, and sucrose were employed.

### 2.3. Bioactive Compounds

#### 2.3.1. Preparation of Leaves Extract

The hot water extract of the entire plant of *L. octovalvis* (Jacq.) P.H.Raven, *C. aconitifolius*, and *C. longirostrata* was prepared as follows: (a) Each plant was separated and cleaned with distilled water, then dried with clean paper towels. (b) The leaves and small branches were separated, and 5 g of each plant were weighed. (c) Three separate beakers of potable water were heated to 90 °C. (d) Then, 5 g of *L. octovalvis* (Jacq.) P.H.Raven, *C. aconitifolius*, and *C. longirostrata* were added to each beaker and allowed to soak for 5 min at 90 °C. (e) The extract was filtered using Whatman n.1 filter paper and stored in amber-colored bottles at 4 °C until use.

#### 2.3.2. Free Amino Nitrogen (FAN)

The ninhydrin colorimetric method was used to determine the FAN content. The standard solution was a glycine solution in water (MB013–500 G). Next, 0.1085 g of pure glycine (C_2_H_5_NO_2_; MW, 75.07; purity, 99%) was dissolved in 100 mL of deionized water in a volumetric flask in accordance with the European Brewery Convention Protocol (Analytica-EBC, 2000). FAN content in this solution was 200 ppm. The 100 ppm, 50 ppm, and 25 ppm solutions were diluted from this stock solution, and a standard curve was created. Following the addition of 1 mL of ninhydrin solution to 5 mL of sample, the mixture was gently stirred for 4–7 min at 80–100 °C. The sample’s amino acids and ninhydrin combined created Ruhemann’s complex, which took on a purple hue. The absorbance at 570 nm was measured after cooling to room temperature in a cold-water bath, and the concentrations of the samples were determined from the standard curve (Lie, 1973) [18].

#### 2.3.3. Total Phenolics

The total phenolic content (TPC) was determined using the Folin–Ciocalteu method. Briefly, 200 μL of each plant infusion was mixed with 50 μL of Folin–Ciocalteu reagent (2 M) and 750 μL of sodium carbonate solution (7.5% *w*/*v*) in a 1.5 mL Eppendorf tube. The mixture was vortexed for 15 s using a Standard Heavy-Duty Vortex Mixer. The tubes were then kept in complete darkness for 2 h and centrifuged at 2500 rpm for 15 min. Next, 200 μL of each sample reaction was transferred to a 96-well microplate, and the absorbance was measured at 620 nm using a microplate reader (Epoch 2, BioTek Instruments Inc., Charlotte, VT, USA). A standard curve was generated using gallic acid (GA) concentrations ranging from 0 to 600 mg/L [19].

#### 2.3.4. Total Flavonoid Content

The total flavonoid content of the plant material was quantified using a colorimetric assay. Specifically, 100 mg of the dried plant material was mixed with 4 mL of distilled water. To this mixture, 0.3 mL of 5% sodium nitrite was added, and after 5 min, 0.3 mL of 10% aluminum chloride was introduced. Following a 6-min incubation period, 2 mL of 1 M sodium hydroxide was incorporated into the solution. Immediately, the mixture was diluted with 3.3 mL of distilled water and thoroughly mixed.

Subsequently, the sample was subjected to centrifugation at 1000× *g* and 4 °C, and 2 mL of the resulting supernatant was collected. The absorbance of the supernatant was measured at 510 nm, using a blank for comparison. Catechin was utilized as a standard to construct the calibration curve. The total flavonoid content of the plant extract was then expressed in milligrams of catechin equivalents per gram of the sample (mg/g) [20].

#### 2.3.5. DPPH Trolox Equivalent

DPPH free radical scavenging activity was determined following the methodology described by García-Becerra et al., 2010 [21]. To estimate the antioxidant capacity of plant infusions, a mixture of 60 µM of a methanolic solution of DPPH (2940 µL) with 60 µL of extract in a polystyrene cuvette was used to measure the absorbance at 515 nm on two occasions: before adding the extract, and 60 min after adding it with a spectrophotometer (Genesys 10S, Annapolis, MD, USA). The standard curve was determined with Trolox, and the results were expressed as a Trolox equivalent in mmol.

#### 2.3.6. Reducing Power µg Ascorbic Acid Equivalents

The method used to determine the reducing power was based on the procedure described by Juntachote et al., 2006 [22], with modifications from Terpinc et al., 2012 [23]. The results are presented as the coefficient of the reducing power (CR). The ability of a compound to act as a reducing agent can indicate its potential antioxidant activity. To conduct the assay, aliquots of the standard and test sample extracts at various concentrations (ranging from 10 to 100 μg/mL) were mixed with 1.0 mL of deionized water, 2.5 mL of phosphate buffer (pH 6.6), and 2.5 mL of potassium ferricyanide (1%). The mixture was then incubated at 50 °C in a water bath for 20 min, followed by cooling. Next, 2.5 mL of trichloroacetic acid (10%) was added, and the mixture was centrifuged at 3000 rpm for 10 min. The upper layer (2.5 mL) of the solution was combined with 2.5 mL of distilled water and 0.5 mL of freshly prepared ferric chloride solution (0.1%). The absorbance was measured at 700 nm using a UV spectrometer (Sytronic double beam-UV-2201). A blank sample without the addition of the extract was prepared, and ascorbic acid at various concentrations (ranging from 10 to 100 μg/mL) was used as the standard [24].

#### 2.3.7. Determination of Antinutrients (Tanins, Alkaloids, Saponins, Oxalate)

The amount of oxalate present in the avocado extract was measured using a modified technique based on Libert and Franceschi’s method from 1987 [25]. A sodium oxalate curve (Sigma Aldrich, 71804, St. Louis, MO, USA) was used as a reference to compare the plant extract. To measure the total tannin content, the spectrophotometric method described by Griffiths and Jones in 1977 was used, utilizing a Tannic acid solution (Sigma Aldrich, 403040, St. Louis, MO, USA) [26]. The saponin content was determined following the approach outlined by Makkar and Becker in 1996, employing a saponin standard (Sigma Aldrich, SAE0073, St. Louis, MO, USA) [27]. Lastly, the alkaloid content was estimated using the spectrometric method developed by Shamsa et al. in 2008, which involves the use of the bromocresol green method [28].

### 2.4. Determination the Inhibition of α-Amylase and α-Glucosidase

The inhibitory activity for *α*-Amylase was obtained following the method of Kazeem et al. [29]. Data were compared by using different extract dilutions and using acarbose (200, 400, 600, 800, 1000, and 1200 μg mL^−1^). Briefly, tubes containing 250 μL acarbose (1 mg mL^−1^) or plant extract along with a sodium phosphate buffer (0.02 M pH 6.9) or 500 μL *α*-Amylase solution in a phosphate buffer (with 0.006 M de NaCl, 13 U mL^−1^) were preincubated at room temperature for 10 min with a 250 μL 1% starch solution (sodium phosphate buffer). Tubes were then boiled for 5 min after adding 1 mL of DNS (method of Miller, 1959) [30] to stop the reaction. As the control blank, a tube without acarbose or plant extract was used. Samples were diluted in 10 mL of distilled water, and absorbance was measured at 540 nm (UNICO S1200, Dayton, NJ, USA). *α*-Amylase inhibition was calculated using Equation (1):

(1) $$\mathrm{Inhibition}\left(\%\right)=\frac{K-(S1-S0)}{K}*100$$

where *K* = absorbance of control blank, *S*1 = absorbance of sample with enzyme, and *S*0 = absorbance of sample without enzyme. Inhibitory activity was expressed as the concentration, allowing half of the maximal inhibitory activity (IC_50_). The IC_50_ value of each extract was determined from the plots of percent inhibition versus inhibitor concentration.

The *α*-Glucosidase test was performed using different extract concentrations as described by Mayur et al. [31]. Rat intestinal acetone powder was mixed with distilled water (10 mg mL^−1^) and centrifuged for 10 min at 10,000 rpm. The assay buffer was 100 mM HEPES at pH 6.8, and the substrate was 2 mM 4-nitrophenyl-*α*-d-glucopyranoside. The assay constituents were added to 96-well microplates in the following order: 100 μL of enzymatic solution (10 mg rat intestinal acetone powder per 1 mL distilled water), then 50 μL of acarbose or seaweed extract or buffer, and finally 50 μL of substrate. Samples were incubated at 25 °C for 2 h. Absorbance was recorded at the beginning and end of the incubation time at 405 nm. *α*-Glucosidase inhibition was calculated using Equation (1). Acarbose (1 mg mL^−1^) was used as a positive control. The inhibitory activity was expressed as the concentration required to obtain half of the maximal inhibitory activity (IC_50_). The extracts’ IC_50_ values were determined from the plots of percent inhibition versus inhibitor concentration.

### 2.5. Determination the Inhibition of Lipase and Pepsin

To conduct the experiment, stock solutions from each herbal product and orlistat were prepared. Orlistat is the only commercial drug known to effectively inhibit pancreatic lipase. The contents of each capsule were dissolved in a 100 mL flask, and these solutions were then diluted to obtain concentrations of 50, 100, 200, 300, and 400 μg/mL. Next, 0.2 mL of each diluted solution was added to separate test tubes. Before proceeding, a stock solution of porcine pancreatic lipase enzyme was prepared at a concentration of 1 mg/mL in a 10% DMSO solution. Subsequently, 0.7 mL of Tris-HCl buffer (pH 7.4, 0.1 M) and 0.1 mL of pancreatic lipase enzyme were added to each test tube. A blank tube was also prepared, containing only 0.9 mL of Tris-HCl and 0.1 mL of pancreatic lipase enzyme without any inhibitor. All the test tubes, including the blank, that contained p-nitrophenyl-butyrate (PNPB) were incubated at 37 °C for 15 min. After the initial incubation, 0.1 mL of PNPB (100 mM in acetonitrile) was added to all the test tubes, including the blank. Another incubation period of 30 min followed. The activity of pancreatic lipase was assessed by measuring the hydrolysis of PNPB to p-nitrophenol at a wavelength of 410 nm using a UV-visible spectrophotometer (Shimadzu-UV-1800, Kyoto, Japan) [32].

Later, the absolute inhibitory activity was calculated using the following equation:

(2) $$Inhibition\left(\%\right)=\frac{\left(B-S\right)}{B}*100\%$$

where *S* refers to the sample absorbance and B to the blank absorbance.

### 2.6. Fourier Transformed Infrared Spectroscopy (FTIR)

After being dried at 40 °C for six hours, the sample was crushed in a coffee grinder. The sample underwent ATR-FTIR analysis (Spectrum 1, Perkin Elmer, Norwalk, VA, USA). Data on molecular spectrum emissions were gathered, adjusted for the air background, and then examined using the Spectrum program (ver. 5.3.0). The spectra were produced using the mid-IR (about 4000–8000) in absorption mode with a resolution of 4 cm^−1^ by 20 scans and a half-band width of 15 cm^−1^. Using published studies (De La Rosa-Millán, 2017; Chávez-Murillo et al., 2018) [33,34] all chemical functional groups were identified.

### 2.7. Statistical Analysis

The obtained results underwent a one-way ANOVA followed by the Tukey multiple comparison test to determine the presence of statistically significant differences. Additionally, a hierarchical cluster analysis was conducted to identify meaningful correlations among the data collected: protein, total flavonoids, free amino nitrogen, IC50 α-Amylase, IC50 α-Glucosidase, lipids, soluble fiber, insoluble fiber, IC50 lipase, ash, reducing power, oxalates, IC50 pepsin, free sugars, DPPH, total phenolics, alkaloids, tanins, and saponins. A hierarchical clustering was applied to the principal components analysis to create a dendrogram.

## 3. Results

### 3.1. Proximal Composition

The proximal composition of *L. octovalvis*, *C. aconitifolius*, and *C. longirostrata* is presented in Table 1, where the major component of *L. octovalvis* are carbohydrates that correspond to the total dietary fiber, with a 65.30 ± 1.83% from which 28.99 ± 0.75% is soluble fiber, 36.31 ± 1.13% is insoluble fiber, and 13.99 ± 0.55% correspond to free sugars. Another major component is protein with 12.70 ± 1.54%, where the free amino nitrogen was also determined. In the case of *C. aconitifolius*, the major component was also carbohydrates, with a 70.27 ± 1.02% from which 25.35 ± 1.38% is soluble fiber, 28.98 ± 1.78 is insoluble fiber, and 15.94 ± 0.80% correspond to free sugars. Comparing all plants, *C. longirostrata* had the highest percentage of carbohydrates (73.70 ± 2.3%), with soluble fiber accounting for 26.34 ± 1.05% of those carbohydrates, 31.25 ± 1.24% representing insoluble fiber, and 16.10 ± 0.20% corresponding to free sugars. Regarding protein, *C. longirostrata* has the largest proportion of protein (14.51 ± 2.04) compared to *L. octovalvis* and *C. aconitifolius*. *C. longirostrata* has the highest concentration of free amino nitrogen (FAN), which is 330.33 ± 12.22 mg/100 g^−1^, followed by *L. octovalvis* with 259.67 ± 4.16 mg/100 g^−1^, and *C. aconitifolius* with 225.83 ± 5.55 mg/100 g^−1^.

### 3.2. Bioactive Compounds

The bioactive compounds of *L. octovalvis*, *C. aconitifolius*, and *C. longirostrata* are presented in Table 2.

*C. longirostrata*, which has the greatest concentration of phenolic compounds (889.66 ± 2.82 mg GAE/100 g), is followed by *C. aconitifolius*, which has a concentration of 740.57 ± 9.43 mg GAE/100 g, and *L. octovalvis*, which has the lowest concentration (652.96 ± 10.58 mg GAE/100 g). In terms of total flavonoid content, *C. longirostrata* similarly has the largest quantity of flavonoids, with 531.53 ± 10.07 mg of chatequin equivalents/100 g, followed by *C. aconitifolius* and *L. octovalvis*, both of which have 346.03 ± 4.28 and 337.00 ± 3.61 mg of chatequin equivalents/100 g, respectively.

The plant with the largest level of DPPH Trolox equivalent was *C. longirostrata* with 4.52 ± 0.22 µMTE/g, followed by *C. aconitifolius* with 4.34 ± 0.12 µMTE/g, and *L. octovalvis* with 2.35 ± 0.06 µMTE/g. Regarding the reducing power in µg ascorbic acid equivalents, *C. aconitifolius* had the largest quantity at 572.41 ± 4.92, followed by *L. octovalvis* at 443.36 ± 2.14, and *C. longirostrata* at 411.26 ± 3.92.

### 3.3. Antinutrients

Plants also contain a category of compounds called antinutrients, which they use for their own defense [35]. These compounds can have antinutrients that can disrupt nutrient absorption, reduce nutrient intake, hinder digestion and utilization, and potentially lead to other negative consequences [35], but they can also have positive effects on health [36]; for example, at lower concentrations, phytic acid, lectins, phenolic compounds, enzyme inhibitors, and saponins have demonstrated the ability to lower blood glucose and/or plasma cholesterol and triacylglycerol levels. Additionally, saponins have been reported to effectively maintain liver function, prevent osteoporosis, and inhibit platelet agglutination [35].

Analyzing compounds such as tannins, alkaloids, saponins, and oxalates reveals that *C. aconitifolius* has the largest quantity of tannins per unit weight (17.58 ± 0.63 mg/g), followed by *C. longirostrata* (15.34 ± 0.14 mg/g), and *L. octovalvis* (14.13 ± 0.14 mg/g). Similar results were found for the alkaloids in terms of the amount of tannins: *C. aconitifolius* had 2.37 ± 0.04 mg/g, *C. longirostrata* had 1.74 ± 0.11 mg/g, and *L. octovalvis* had 1.12 ± 0.14 mg/g. *C. aconitifolius* (5.49 ± 0.21 mg/g) had the greatest concentration of saponins, followed by *C. longirostrata* (4.42 ± 0.23 mg/g) and *L. octovalvis* (2.26 ± 0.30 mg/g). *C. aconitifolius* had the greatest concentration of oxalates (8.91 ± 0.48 mg/g), followed by *L. octovalvis* (4.41 ± 0.22 mg/g) and *C. longirostrata* (3.32 ± 0.12 mg/g). The information is presented in Table 3.

### 3.4. α-Amylase Inhibition

The ability to inhibit α-Amylase was found to vary among *L. octovalvis*, *C. aconitifolius*, and *C. longirostrata* against the positive control, acarbose. Table 4 presents the results of the α-Amylase inhibition assay conducted on three plant samples (*L. octovalvis*, *C. aconitifolius*, and *C. longirostrata*) and Acarbose at different concentrations. The inhibitory effects of the plants and drug were expressed as percentages of dry matter. The results demonstrate that the IC_50_ values for *L. octovalvis*, *C. aconitifolius*, and *C. longirostrata* were 519.40 mg/mL, 471.39 mg/mL, and 585.12 mg/mL, respectively, while Acarbose had an IC_50_ value of 338.49 mg/mL. The IC_50_ value refers to the concentration at which a 50% inhibition of α-Amylase activity is achieved.

It is evident that the inhibition of α-Amylase activity becomes more pronounced as the concentration of the samples increases. Among the tested samples, *L. octovalvis* exhibits the lowest inhibition activity, with an IC_50_ value of 519.40 mg/mL, while *C. aconitifolius* demonstrates the highest inhibition activity, with an IC_50_ value of 471.39 mg/mL. These findings indicate that *C. aconitifolius* is the most potent inhibitor of α-Amylase activity among the samples examined.

**Figure 1:**
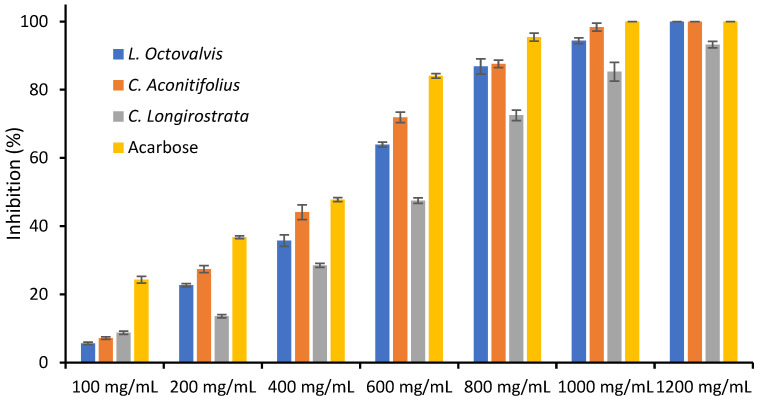
α-Amylase inhibition of *L. octovalvis*, *C. aconitifolius*, and *C. longirostrata* plant extracts.

**Figure A1:**
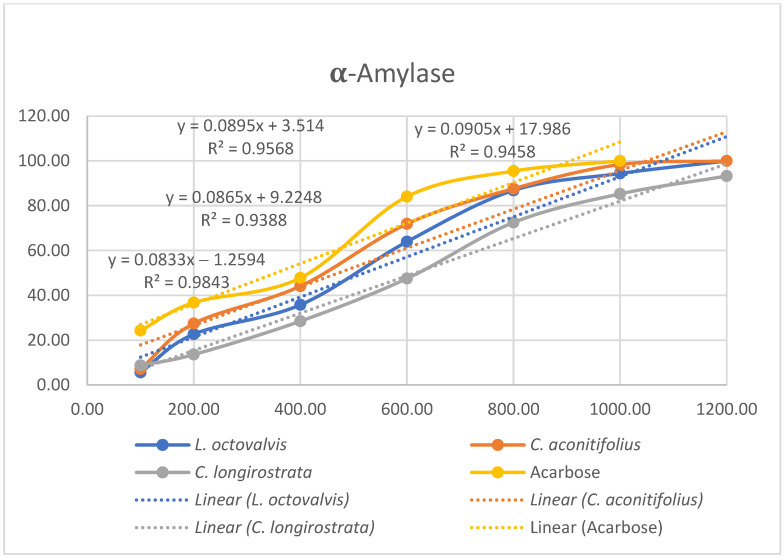
The α-Amylase inhibition of *L. octovalvis*, *C. aconitifolius*, and *C. longirostrata* plants expressed as a percentage of dry matter. The data is presented in line charts with trendlines.

Figure 1 depicts the percentage of α-Amylase inhibition of the *L. octovalvis*, *C. aconitifolius*, and *C. longirostrata* plants (dm). The graph shows an increasing trend in inhibition percentage with increasing extract concentration. It is evident that there is a significant difference between the groups, from concentrations ranging from 100 mg/mL to 600 mg/mL, as indicated by the non-overlapping error bars. At a concentration of 800 mg/mL, there is no significant difference between *L. octovalvis* and *C. aconitifolius*, while there is a significant difference between *C. longirostrata* and acarbose. From 1000 mg/mL, there is no significant difference between *L. octovalvis*, *C. aconitifolius*, and acarbose, but there is a significant difference with *C. longirostrata*. In Figure A1, the data are presented in line charts with trendlines.

### 3.5. Glucosidase Inhibition

Table 5 shows the α-Glucosidase inhibition of three different plants (*L. octovalvis*, *C. aconitifolius*, and *C. longirostrata*) at various concentrations of plant extract (100–1200 mg/mL) and acarbose. As can be seen, if the concentration of the plant extract increased, the percentage of α-Glucosidase inhibition also increased for all three plant species. Finally, the IC_50_ values for each plant extract and Acarbose were also reported, indicating the concentration required to inhibit 50% of the α-Glucosidase activity.

**Figure 2:**
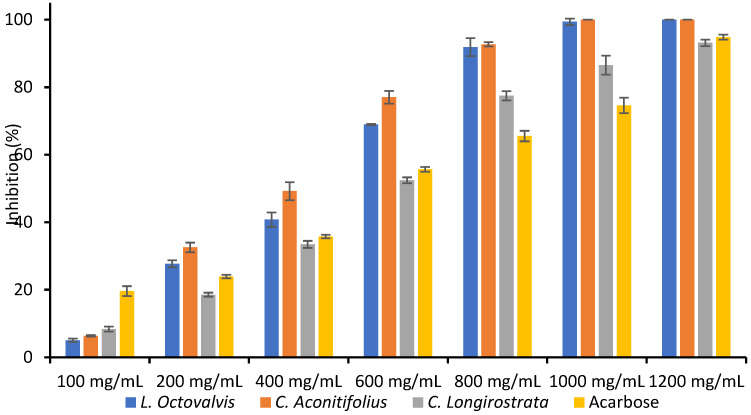
α-Glucosidase inhibition of *L. octovalvis*, *C. aconitifolius*, and *C. longirostrata* plant extracts.

Figure 2 shows the concentrations between 100–600 mg/mL, and there was a significant difference in α-Glucosidase inhibition between the three plant species, as indicated by the non-overlapping error bars.

At concentrations above 800 mg/mL, there was no significant difference in α-Glucosidase inhibition between *L. octovalvis* and *C. aconitifolius*, but there was a significant difference between both of these plants and *C. longirostrata*. At 1000 mg/mL, there was no significant difference in α-Glucosidase inhibition between *L. octovalvis*, *C. aconitifolius*, and acarbose, but there was a significant difference with *C. longirostrata* (Figure 2).

### 3.6. Lipase Inhibition

In Table 6 it is shown the Lipase inhibition of three different plants (*L. octovalvis*, *C. aconitifolius*, and *C. longirostrata*) at various concentrations of plant extract (100–1200 mg/mL) and Orlistat as control. In the observed data, it is evident that elevating the concentration of the plant extract resulted in a proportional increase in the inhibition of Lipase activity across all three plant species. 

**Figure 3:**
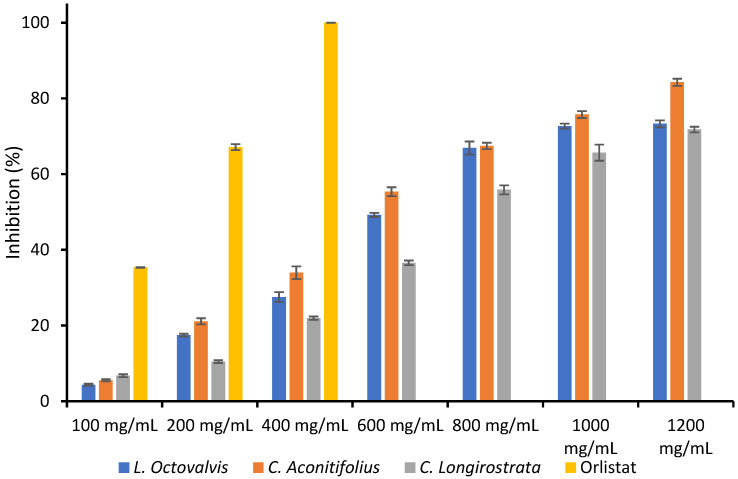
Lipase inhibition of *L. octovalvis*, *C. aconitifolius*, and *C. longirostrata* plant extracts.

In Figure 3 it is displayed the percentage of lipase inhibition for the *L. octovalvis*, *C. aconitifolius*, and *C. longirostrata* plants at various concentrations, as well as for the positive control Orlistat. As the concentration of the plant samples increases, so does the percentage of lipase inhibition. At the highest concentration of 1200 mg/mL, all three plant samples exhibit substantial lipase inhibition, with *C. aconitifolius* showing the highest percentage (84.27 ± 0.94). Orlistat, the positive control, completely inhibits lipase activity at all concentrations. In general, the plant samples differ significantly in their lipase inhibitory activity, with *C. aconitifolius* consistently displaying greater inhibition than the other two plant samples. 

### 3.7. Pepsin Inhibition

Table 7 displays the outcomes of the pepsin inhibition test performed on the three plant specimens (*L. octovalvis*, *C. aconitifolius*, and *C. longirostrata*) and a positive control (protease inhibitor cocktail) at varying concentrations (ranging from 100 to 1200 mg/mL) and the inhibitory effects were expressed as percentages of dry matter. The findings indicate that the higher the concentration of the plant extract, the greater the percentage of pepsin inhibition. The IC_50_ values for *L. octovalvis*, *C. aconitifolius*, and *C. longirostrata* were 910.94 mg/mL, 1039.93 mg/mL, and 823.02 mg/mL, respectively, which refers to the concentration at which a 50% inhibition of pepsin activity occurs. At 200 mg/mL, *C. aconitifolius* and the protease inhibitor cocktail showed complete inhibition of pepsin activity, while *L. octovalvis* and *C. longirostrata* did not achieve full inhibition at any of the tested concentrations. It is crucial to note that the protease inhibitor cocktail was employed as a positive control and not as a sample for comparison with the plant extracts.

The plant samples show significant differences in their inhibitory effects at various concentrations. For instance, *C. longirostrata* has a higher inhibitory effect than *L. octovalvis* and *C. aconitifolius* at 100 mg/mL. However, at 200 mg/mL, *C. aconitifolius* exhibits significantly higher inhibition than the other two plant samples, achieving complete inhibition. Neither *L. octovalvis* nor *C. longirostrata* show complete inhibition at any tested concentration.

**Figure 4:**
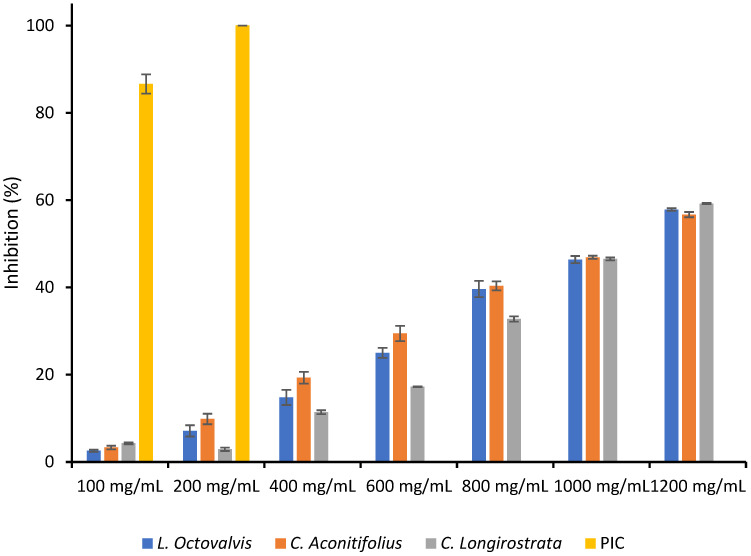
Pepsin inhibition of *L. octovalvis*, *C. aconitifolius*, and *C. longirostrata* plant extracts.

Furthermore, it is important to note that the protease inhibitor cocktail has a much stronger inhibitory effect on pepsin than the plant samples at all concentrations, indicating its high effectiveness as an inhibitor (Figure 4).

### 3.8. FTIR

**Figure 5:**
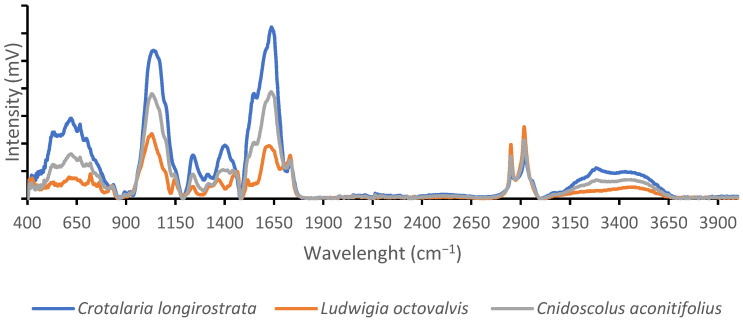
*L. octovalvis*, *C. aconitifolius*, and *C. longirostrata* FTIR spectrum.

Figure 5 shows the spectrum determined by the FTIR of *L. octovalvis*, *C. aconitifolius*, and *C. longirostrata.*

### 3.9. Statiscal Analysis

**Figure 6:**
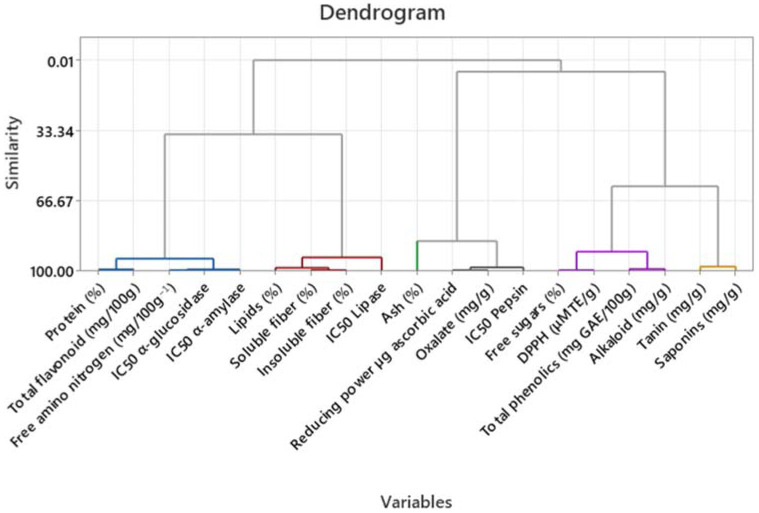
Hierarchical cluster analysis of *L. octovalvis*, *C. aconitifolius*, and *C. longirostrata* components.

With the objective of comprehensively analyzing the various evaluated parameters in this research, a hierarchical clustering analysis was done. Significant correlations are illustrated in Figure 6. The analysis resulted in the identification of five distinct clusters, each closely associated with the IC_50_ values of enzyme inhibition. The first cluster comprises proteins, flavonoid content, FAN, IC_50_ α-Amylase, and IC_50_ α-Glucosidase. The second cluster includes lipids, soluble fiber, insoluble fiber, and IC_50_ lipase. In the third cluster, we find Ash, FRAP, oxalates, and IC_50_ pepsin. The fourth cluster contains free sugars, DDPH, total phenolics, and alkaloids, while the fifth cluster consists of tannins and saponins.

## 4. Discussion

### 4.1. Proximal Composition

One of the objectives of this study was to determine the chemical composition of *L. octovalvis* (Jacq.) P.H.Raven, *C. aconitifolius*, and *C. longirostrata* to gain a better understanding of the nutritional properties of each plant. Oyagbemi et al. (2011) conducted a proximate analysis of *C. aconitifolius*, revealing protein content at 14.61%, fat at 2.58%, ash at 9.98%, and fiber at 9.88%. When comparing our results solely with the parameters examined by Oyagbemi et al., we found that the protein content reported in our study (12.52%) was lower than their finding (14.61%). Conversely, our data showed higher fat content (5.67%) compared to theirs (2.58%), and lower ash content (6.66%) in contrast to their value of 9.98%. Notably, our fiber content (54.33%) was substantially higher than their reported value (9.885%). These differences might be influenced by factors such as sample variability, analytical methodologies, environmental conditions, or genetic diversity among the studied *C. aconitifolius* specimens. Hence, when comparing nutritional data from different studies, it is crucial to consider these factors for accurate interpretation and understanding of the observed variations [37].

For *C. longirostrata*, a comparative analysis was conducted with a plant of the same genus, *Crotalaria retusa*, as reported in the study by Alalade in 2019. The findings revealed notable differences between the two datasets. Specifically, the protein content reported in Alalade et al. (2019) (18.00%) was higher than that observed in our research (14.51%). Likewise, the ash content reported by Alalade et al. (2019) (7.00%) significantly exceeded the value found in our research (0.71%). Furthermore, the insoluble fiber content in our research (31.25%) was substantially higher than the value reported in Alalade et al. (2019) (16.75%) [38].

These observed variations in protein, ash, and insoluble fiber content may be attributed to several factors, such as differences in plant samples, analytical methods, environmental conditions, or genetic factors that influence the nutritional composition of *C. longirostrata*. Therefore, it is imperative to consider these factors when comparing nutritional data from various studies to ensure accurate interpretation and comprehension of the discrepancies observed.

A comparison was conducted between *L. octovalvis* (Jacq.) P.H.Raven and *Ludwigia grandiflora* in a study carried out by Oku et al. in 2020. The choice of Ludwigia grandiflora was due to the unavailability of data for *L. octovalvis* in the existing literature. Our experimental findings indicate that *L. octovalvis* exhibits a higher protein content (12.70%) compared to the value reported by Oku et al. (10.31%). Additionally, our results show that *L. octovalvis* has a lower ash content (6.01%) in contrast to the value presented by Oku et al. (7.89%). Moreover, our data demonstrate a significantly higher level of insoluble fiber in *L. octovalvis* (36.31%) compared to Oku et al.’s findings (19.94%) [39]. These disparities between the datasets for *L. octovalvis* should be interpreted with consideration of potential factors such as the comparison with different species that have the same genus, sample source, analytical methods, and experimental procedures, that could contribute to the observed differences.

### 4.2. Bioactive Compounds

It is important to emphasize that the plant extracts in this study were prepared using hot water instead of an alcoholic solvent. While many research studies commonly employ alcoholic extracts to comprehensively analyze plant composition, the objective of our investigation is to gain insights into the plants from a more realistic perspective, particularly when considering edible plants that are naturally consumed without the extraction of compounds through alcoholic solvents such as methanol or ethanol. The selection of an appropriate solvent and extraction technique is crucial for effectively extracting biologically active compounds from medicinal plants. The polarity of solvents plays a critical role in separating specific compounds with diverse structures and physicochemical properties. Water, ethanol, and glycerol are well-established solvents that are recognized and approved for use in pharmaceutical formulations [40].

According to Shopska et al. (2019), “free amino nitrogen” (FAN) refers to nitrogen-containing molecules that are readily available, such as amino acids, peptides, and ammonium ions [41]. The aim of this study was to determine the form in which protein is present, and the results suggest that it contains free amino acids. Amino acids are crucial constituents of plants and serve as the basic building blocks for proteins, the main nitrogen carriers, and signaling molecules. Plant amino acids are produced through three different processes: absorption by roots, reduction in nitrates, and breakdown of ammonium, as explained by Guo et al. (2021) [42]. Due to the scarce information on the free amino nitrogen content in *L. octovalvis*, *C. aconitifolius*, and *C. longirostrata*, an analysis of FAN content was carried out. When comparing the free amino nitrogen content of *L. octovalvis*, *C. aconitifolius*, and *C. longirostrata*, it can be observed that *C. longirostrata* has the highest content, with a mean value of 330.33 ± 12.22 mg/100 g^−1^, followed by *L. octovalvis*, with a mean value of 259.67 ± 4.16 mg/100 g^−1^, and finally, *C. aconitifolius*, with a mean value of 225.83 ± 5.55 mg/100 g^−1^. Free amino nitrogen includes amino acids, peptides, and ammonium ions and is an important component of plant protein.

Phenolics represent the most extensive category of phytochemicals, playing a pivotal role in the majority of antioxidant activity found within plants and plant-derived products [43]. The quantification of total phenolics is of significant importance as phenolic compounds serve as vital constituents of plants, exhibiting redox properties that contribute to their antioxidant activity [44]. Compared to a study made by Yakob et al. in 2012, the total phenolic compounds found in *L. octovalvis* using the same methodology was 264.76 ± 0.23 µg GAE/g [45]. Another study conducted by Lin et al. in 2017 found that the polyphenol content was 146.3 ± 3.1 mg GAE/g [11], which is lower than the quantity obtained in this research, 652.96 ± 10.58 mg GAE/g. This could be because of a difference in the solvents used (methanol vs. warm water). In the case of *C. aconitifolius*, there are few research studies reporting the total phenolic content. In a study conducted by Godínez-Santillán et al. in 2019 [46], it was found that the total phenolic content was 59.2 ± 2.3 mg CE/g in a methanol:water (50:50) extract. In comparison, in our research, we found a content of 740.57 ± 9.43 mg GAE/100 g, which differs significantly; this was also possibly due to the different solvents used. A study conducted by Jiménez Aguilar and Grusak in 2015 [47] on *C. longirostrata* revealed that the total phenolic compounds ranged from 2.68 ± 0.14 mg GAE/g FW to 3.38 ± 0.24. In comparison, our research found that the total phenolics were 889.66 ± 2.82 mg GAE/100 g. This differs because the result of Aguilar and Grusak’s study is expressed on a fresh weight basis, and ours is expressed on a dry weight basis. Additionally, this difference in results could be attributed to variations in the extraction method employed, as well as the solvent; the previous study utilized 2.5 mL of 90% methanol at 90 °C for 2 h in a water bath, with intermittent vortexing every 30 min. On the other hand, we used hot water at 90 °C for 5 min.

Flavonoids, the largest subgroup among naturally occurring phenolic compounds, are present in various plant components, existing in their unbound state or as glycosides [43]. Flavonoids, which are widely distributed in plants, have been extensively utilized in traditional herbal remedies. They constitute essential constituents of our diet and are predominantly present in edible plant parts. Flavonoids can be found in various sources such as fruits, vegetables, grains, tree bark, stems, tea, and wine. When addressing complex chronic ailments, conventional treatment methods often rely on polypharmacy, involving the use of multiple medications. It is crucial to recognize that herbal medicines are intricate mixtures comprising diverse elements, both major and minor, and exhibiting multiple targets and processes, thus possessing complex chemical characteristics [48].

In our investigation, the total flavonoid content in *L. octovalvis* was determined to be 337.00 ± 3.61 mg of catechin equivalents per 100 g. However, another study conducted by Pandey et al. in 2023 reported a relatively lower total flavonoid content of 43.9 mg of catechin equivalents per gram. Pandey et al., 2023, employed an extraction method utilizing a mixture of ethanol and water (70:30 *v*/*v*) [49]. In the case of *C. aconitifolius*, the total flavonoid content was determined to be 346.03 ± 4.28 mg of catechin equivalents per 100 g, as indicated in a study conducted by Padilla-Camberos. Another investigation by Padilla-Camberos revealed a flavonoid content of 154.23 ± 3.35 mg of catechin equivalents per milliliter, utilizing an ethyl acetate extract [50]. The observed significant difference in results could potentially be attributed to the use of different solvents. For *C. longirostrata*, we determined that the total flavonoid content was 531.53 ± 10.07 mg of catechin equivalents per 100 g. However, Mendez-Lopez et al. (2022) reported a flavonoid content of 20.8–35.3 mg/g [51], demonstrating significant discrepancies. These variations can also be attributed to the choice of solvents, specifically hexane in this case.

Trolox, a synthetic analog of α-tocopherol that can dissolve in water, is commonly used as a standard compound to evaluate the Trolox equivalent antioxidant capacity (TEAC) of various food samples. α-tocopherol is widely recognized as the most potent form of vitamin E. The TEAC comparison is based on Trolox’s ability to demonstrate antioxidant activity similar to other natural substances found in food. With its high solubility of approximately 3 mg/mL at neutral pH levels, Trolox is suitable for conducting various antioxidant assays. This allows for the measurement of the antioxidant capacity of food samples by comparing their activity to the known antioxidant capacity of Trolox. Among the most commonly employed methods, a 1,1-diphenyl-2-picrylhydrazyl (DPPH) assay is frequently performed, which involves evaluating the scavenging ability of antioxidants against the DPPH radical [52].

To validate the findings of this study, a comprehensive review of the literature was conducted to examine previous research on the utilization of 1,1-diphenyl-2-picrylhydrazyl (DPPH) in *L. octovalvis*. Although no studies utilizing water extracts were discovered, a study conducted by Yakob et al. in 2012 investigated the application of DPPH using various alcoholic extracts. The outcomes indicated that the methanol extract exhibited the highest DPPH content (1080.84 ± 6.07 μM TE/mg d.w), followed by the ethyl acetate extract (301.48 ± 8.92 μM TE/mg d.w). Conversely, the chloroform extract displayed a lower DPPH content (113.04 ± 1.58 μM TE/mg d.w), while the n-hexane extract exhibited the lowest DPPH content (91.00 ± 0.31 μM TE/mg d.w) [45]. These findings underscore the significant influence of solvent selection on the DPPH content present in the extracts. In our investigation, a DPPH value of 2.35 ± 0.06 µg ascorbic acid equivalents were determined, thus confirming the substantial impact of solvent choice on the DPPH content.

In the case of *C. aconitifolius*, an investigation conducted by Godínez-Santillán et al. in 2019 was discovered, wherein this plant and its DPPH content were examined. It was observed that *C. aconitifolius* exhibited a content of 140 ± 6 in the methanol–water (50:50) extract, while the ethanol–water (50:50) extract displayed a content of 102 ± 6 [46]. These findings diverge significantly from our own research, similar to the DPPH content of *L. octovalvis*. Our investigation revealed a DPPH content of 4.34 ± 0.12, representing a substantial difference. This discrepancy may be ascribed to the variances in methodology and the differing solvents employed (methanol, ethanol vs. warm water).

In the case of *C. longirostrata*, a comparison was conducted with a previous investigation carried out on *Crotalaria pallida* by Alam et al. in 2014. Since no specific studies were found on the same plant species, *Crotalaria pallida* was used as it belongs to the same genus [53]. Comparing the DPPH content between *Crotalaria pallida* and *C. longirostrata* reveals a significant difference in their antioxidant capacities. The study conducted on *Crotalaria pallida* reported a DPPH content of 37.60 μg/mL. In contrast, our investigation on *C. longirostrata* found a considerably lower DPPH content of 4.52 ± 0.22 µM TE/g. This discrepancy suggests that *Crotalaria pallida* exhibits a higher antioxidant capacity, as indicated by its higher DPPH content, compared to *C. longirostrata*. The variation in DPPH content could be attributed to factors such as plant species, extraction methods, or other environmental factors. Further research is needed to elucidate the underlying reasons for this divergence.

Reducing power (FRAP) assay is an efficient and cost-effective technique for directly assessing the overall antioxidant activity of electron-donating antioxidants in a given sample. It involves the reduction of ferric ions (Fe^3+^) to ferrous ions (Fe^2+^), which triggers a color change, serving as an indicator of the reaction. The method is known for its simplicity, rapidity, and affordability [47]. Based on our investigation we can conclude that *C. aconitifolius* has the highest FRAP value (572.41 ± 4.93 µg ascorbic acid equivalents), indicating stronger total antioxidant activity. *L. octovalvis* has a lower FRAP value (443.36 ± 2.14 µg ascorbic acid equivalents), suggesting a moderate level of antioxidant activity. Finally, *C. longirostrata* exhibits the lowest FRAP value (411.26 ± 3.92 µg ascorbic acid equivalents), indicating relatively weaker antioxidant activity compared to the other two plants.

To validate the accuracy of our obtained results, a comprehensive literature search was conducted to identify relevant studies that investigated the FRAP (Ferric Reducing Antioxidant Power) content of the same plant species. Although no studies utilizing water extracts were found, a notable study by Yakob et al. in 2012 provided insights into the FRAP content of *L. octovalvis* using various alcoholic extracts. Their findings indicated that the methanol extract exhibited the highest FRAP content (1256.88 ± 5.38 μM TE/mg d.w), followed by the ethyl acetate extract (253.45 ± 5.97 μM TE/mg d.w). In contrast, the chloroform extract displayed a relatively lower FRAP content (136.83 ± 3.48 μM TE/mg d.w), while the n-hexane extract demonstrated a similar FRAP content (142.04 ± 3.53 μM TE/mg d.w) [45]. These results strongly suggest that the choice of solvent for extraction significantly influences the FRAP content of the extracts, with methanol exhibiting the highest antioxidant capacity.

Upon comparing our investigation’s results (443.36 ± 2.14 µg ascorbic acid equivalents) with the findings from the study conducted by Yakob et al. (2012), a notable discrepancy in the FRAP content of the plant extracts became evident. Our investigation reported a significantly higher FRAP content of 443.36 ± 2.14, which contrasts with the values obtained in the Yakob et al. study [45]. This difference may be attributed to various factors, such as variations in plant samples, extraction methods, or discrepancies in laboratory techniques employed. Further analysis and exploration are required to determine the specific reasons underlying this discrepancy.

In the case of *C. aconitifolius*, research conducted by Godínez-Santillán et al. in 2019 was discovered [46]. In this investigation, the FRAP content of *C. aconitifolius* was determined, revealing a content of 133 ± 1 μmol TE/g DW in the methanol–water (50:50) extract, and 86 ± 2 μmol TE/g DW in the ethanol–water (50:50) extract. However, in our own research, we found a content of 572.41 ± 4.93 µg ascorbic acid equivalents. These discrepancies can primarily be attributed to the use of water-only extraction in our study, while their investigation involved a combination with an alcohol extract, leading to potential differences in the measured quantities.

In the case of *C. longirostrata*, no FRAP content reports were available. However, in a study conducted by Govindappa et al. in 2011, the FRAP content of the water extract from *Crotalaria pallida* was reported as 1323.08 ± 0.03 μmol/L [54]. In contrast, our research on *C. longirostrata* determined a FRAP value of 411.26 ± 3.92 µg ascorbic acid equivalents. These results indicate a significant difference in the FRAP content between the two studies. Various factors could contribute to this disparity, such as variations in the specific plant species, differences in methodology, variations in the growth conditions or harvesting period, and the choice of solvent for extracting and quantifying the FRAP content.

## 5. Antinutrients

Tannins, which are bitter and astringent plant compounds classified as polyphenols, possess the capability to bind or cause proteins, as well as other organic substances such as amino acids and alkaloids, to form precipitates. These compounds are frequently present in food items and have been observed to inhibit the functions of various enzymes, including trypsin, chymotrypsin, Amylase, and lipase. Additionally, tannins can diminish the protein quality of foods and disrupt the absorption of dietary iron. It has been discovered that tannins can impede the digestion process by exhibiting anti-Amylase activity [55].

According to our research, *C. aconitifolius* has the highest tannin content, with 17.58 ± 0.63 mg/g. This is followed by *C. longirostrata* with 15.34 ± 0.14 mg/g, and *L. octovalvis* with the lowest tannin content of 14.13 ± 0.14 mg/g. However, it is important to note that the values we provide are approximate and include a margin of error (indicated by the ± symbol). These values suggest the average tannin content per gram of dried plant material, but variations may occur due to factors such as growing conditions, plant age, and analytical methods used.

Alkaloids are diverse chemical compounds synthesized by plants, often found as the salts of plant acids. They are present in approximately 15 to 20 percent of vascular plants and consist of small organic molecules with carbon rings and side chains, frequently containing nitrogen atoms [55]. Alkaloids have vital roles in both human medicine and the natural defense mechanisms of organisms. They contribute to about 20% of known plant secondary metabolites, providing protection against predators and regulating plant growth. Moreover, alkaloids possess therapeutic properties and are recognized for their anesthetic, cardioprotective, and anti-inflammatory effects [56]. It is important to note that alkaloids are classified as anti-nutrients as they can interfere with the nervous system, leading to disruptions or excessive enhancement of electrochemical transmission [55] From the research conducted, it was found that *C. longirostrata* has the highest alkaloid content with 2.37 ± 0.04 mg/g. *C. aconitifolius* has a higher alkaloid content compared to *L. octovalvis*, with values of 1.74 ± 0.11 mg/g and 1.12 ± 0.14 mg/g, respectively.

Saponins are a diverse group of natural compounds found in various plants that have the ability to produce foam. They are classified as secondary compounds and are widely present in the plant kingdom. Saponins are typically non-volatile and have surfactant properties. They have been found to reduce the availability of nutrients and decrease enzyme activity, affecting the digestion of proteins by inhibiting enzymes such as trypsin and chymotrypsin. However, saponins have also gained attention due to their potential positive effects on human health. Recent evidence suggests that saponins possess properties such as hypocholesterolemia (lowering cholesterol levels), immunostimulatory (boosting the immune system), and anticarcinogenic properties (preventing or inhibiting the growth of cancer cells) [55]. Thanks to our research, it was found that when comparing the saponin levels, *C. aconitifolius* has the highest saponin content at 5.49 ± 0.21 mg/g. *C. longirostrata* has a slightly lower saponin content of 4.42 ± 0.23 mg/g, while *L. octovalvis* has the lowest saponin content among the three plants at 2.26 ± 0.30 mg/g.

Oxalates are natural compounds found in certain vegetables, including spinach, chard, beets, and rhubarb. They are considered antinutrients and have strong acidic properties. Oxalates can form soluble salts with minerals such as sodium and potassium, as well as insoluble salts with calcium, iron, or zinc. Consuming foods high in oxalates has traditionally been associated with an increased risk of developing kidney stones, based on human studies. When assessing oxalate content in foods, it is important to recognize that soluble oxalates have a greater impact on bioavailability and the risk of stone formation compared to insoluble oxalates [36]. Comparing the oxalate levels obtained, *C. aconitifolius* has the highest oxalate content at 8.91 ± 0.48 mg/g. *L. octovalvis* has a lower oxalate content of 4.41 ± 0.22 mg/g, while *C. longirostrata* has the lowest oxalate content among the three plants at 3.32 ± 0.12 mg/g.

### 5.1. α-Amylase Inhibition

α-Amylase is an essential enzyme synthesized by the pancreas and salivary glands that plays a vital role in the digestion of starch and glycogen. It is found in various organisms, including microorganisms, plants, and higher organisms. This enzyme is responsible for the initial breakdown of starch into smaller oligosaccharides, such as maltose, maltotriose, and branched α-(1–6) and α-(1–4) oligoglucans. These smaller molecules are subsequently further degraded into glucose by α-Glucosidases, facilitating their absorption into the bloodstream. By inhibiting the activity of α-Amylase, the increase in postprandial glucose levels can be controlled by slowing down the hydrolysis and absorption of carbohydrates. Furthermore, the antioxidant properties of plants are often highlighted for their beneficial effects in preventing diabetes and other chronic diseases [57].

A literature review was conducted to explore previous studies on α-Amylase inhibition in *L. octovalvis.* One study by Binh et al., 2016, was identified, in which they investigated α-Amylase inhibition but did not observe any inhibitory effects, despite conducting tests with ethanol and water for the plant extraction [4]. In contrast, our own study found a significant inhibition of 519.40. These disparities could be attributed to variations in the experimental methodologies employed in both studies, as well as the inherent variability in the composition of the parts of the plant used. Factors such as harvesting season, and geographical region of cultivation can influence the chemical profile of the plant, thus potentially impacting its α-Amylase inhibitory activity.

*C. aconitifolius* was studied by Ramos-Gómez, where an inhibition of 0.094 IC_50_ in mg/mL was found for the α-Amylase [58]. However, in our research, we found an IC_50_ of 471.39 mg/mL, which can be attributed to differences in the methodology employed. Both studies used the same water extraction method, but they differed in the boiling time. While Ramos-Gómez boiled the sample for 20 min, we only performed a 5 min infusion.

Due to *C. longirostrata* being a poorly studied plant, there is limited information available regarding studies on its enzymatic inhibition. However, a comparison was made with research conducted by Anwar et al. in 2022 [59], where a plant called *Crotalaria burhia* Buch.-Ham, belonging to the same genus as *C. longirostrata*, was studied. In this study, the inhibitory activity of α-Amylase was measured, and it was found that with methanol extracts, an IC_50_ inhibition of 0.63 mmol/g was observed. This may suggest that the enzymatic inhibition is different to that found in our study, where we found that *C. longirostrata* exhibited an IC_50_ inhibition of 585.12 mg/mL. Although the studied plants are not the same, it can help guide us in assessing the accuracy of our results because both plants belong to the same genus. However, there is no available information with which we can directly compare the results obtained in our research.

### 5.2. α-Glucosidase Inhibition

Slowing down the increase in blood sugar after consuming carbohydrates can be achieved by inhibiting a specific enzyme. This enzyme is located in the membrane of the small intestine’s epithelium. Its main function is to assist in the absorption of glucose by catalyzing the breakdown of oligosaccharides into easily absorbable monosaccharides. When α-Glucosidase in the intestine is inhibited, the hydrolysis of oligosaccharides is reduced, causing the process of carbohydrate digestion to occur in the lower portion of the small intestine. As a result, the overall absorption rate of glucose into the bloodstream is delayed. This approach has proven to be highly effective in reducing the rise in blood glucose levels after a meal and can help prevent the development of complications associated with diabetes [57].

Based on these values, it can be observed that *C. aconitifolius* has the lowest IC_50_ value (433.78 mg/mL), indicating a stronger inhibition of α-Glucosidase activity. *L. octovalvis* has a slightly higher IC_50_ value (480.50 mg/mL), suggesting a moderate level of α-Glucosidase inhibition. On the other hand, *C. longirostrata* (579.71 mg/mL) and Acarbose (571.61 mg/mL) have higher IC_50_ values, indicating weaker inhibition compared to the other samples. It is important to note that these values represent the inhibitory concentration required, with lower values indicating stronger inhibition.

Regarding *L. octovalvis*, Ramirez et al. (2012) found an inhibition with an IC_50_ value of 0.202 mg/mL [10], while Morales et al. (2018) found an inhibition with an IC_50_ value of 0.700 mg/mL [14]. In our study, we found an inhibition with an IC_50_ value of 480.50 mg/mL. These differences are attributed to the use of different hydroalcoholic extracts, as we did not use alcohol in our research. *C. aconitifolius* was studied by Ramos-Gómez, where an inhibition of 0.089 ± 0.7 IC_50_ in mg/mL was found for the α-Glucosidase [58]. However, in our research, we found an IC_50_ of 433.78 mg/mL, which can be attributed to differences in the methodologies employed. Both studies used the same water extraction method, but they differed in the boiling time. While Ramos-Gómez boiled the sample for 20 min, whereas we only performed a 5 min infusion.

To make a comparison in the literature on *C. longirostrata*, a study conducted by Sut et al. in 2020 was used [60]. In this investigation, *Crotalaria retusa* and its inhibitory power were studied. The comparison was made with this plant because it belongs to the same genus as *C. longirostrata*. The study concluded that α-Glucosidase inhibition was not active, which differs from our findings. In our research, we found an inhibition of 579.71 mg/mL. This difference may be due to the fact that it is not exactly the same plant. Although they belong to the same genus, their chemical composition is different, which may affect α-Glucosidase inhibition and explain the contrasting results.

### 5.3. Lipase Inhibition

By binding to the active part of lipase in the stomach and small intestine, lipase inhibitors alter the structure of both stomach and trypsin enzymes, leading to the inhibition of their catalytic activity. Consequently, the breakdown of lipids, such as triglycerides, is diminished. This reduction in hydrolysis hampers the digestion and absorption of dietary lipids, ultimately resulting in decreased accumulation of adipose tissue. These effects contribute to the control and treatment of obesity [61].

*L. octovalvis* was studied by Morales et al. in 2018, and the dry extract displayed an IC_50_ value of 0.480 mg/mL [14]. In our study it was found that *L. octovalvis* has an IC_50_ value of 697.17 mg/mL, and this difference can be attributed to the extraction method and the solution, which was 60% ethanol. It is also important to mention that these differences can be due to the parts of the plant used or the plant itself, which is related to the harvest season and its growing conditions.

*C. aconitifolius* was studied by Ramos-Gómez, where an inhibition of 0.065 ± 0.000 IC_50_ in mg/mL was found [58]. However, in our research, we found an IC_50_ of 627.84 mg/mL, which can be attributed to differences in the methodology employed. Both studies used an aqueous extraction, but they differed in the boiling time. While Ramos-Gómez boiled the sample for 20 min, we conducted a 5 min infusion. Due to the lack of information on *C. longirostrata*, a comparison will be made with *Crotalaria pumila* Ort., which belongs to the same Fabaceae family. In an investigation conducted by Villa-Ruano et al. in 2013, it was found that *Crotalaria pumila* has an IC_50_ inhibition of 0.075 ± 0.002 μg/mL [61]. In order to determine the lipase inhibition, an ethanol extraction was performed for 15 days. This differs from our research because we found that *C. longirostrata* has an IC_50_ inhibition of 677.27 mg/mL. This difference could be due to the applied methodology as well as the type of extraction used.

### 5.4. Pepsin Inhibition

Pepsin, which is an enzyme categorized as an aspartic protease [7], facilitates the breakdown of proteins and peptides by releasing amino acids and small peptides. It is predominantly found in significant amounts in the digestive tracts of humans. Certain protease inhibitors derived from plants have the potential to hinder the digestive process of dietary proteins in the human gastrointestinal system by inhibiting trypsin and chymotrypsin. However, scientific evidence also suggests that these inhibitors offer favorable effects in the gut, including anti-inflammatory and chemo-preventive actions, both in laboratory studies and in living organisms [8]. Considering that the inhibition of pepsin has not been thoroughly studied, there are not many studies in the literature to compare the plants studied in this paper. Therefore, a comparison between them will be made based on the inhibition found in the IC_50_ values. The values found represent the concentration of a substance required to inhibit the activity of the protease enzyme by 50%. The lower the IC_50_ value, the more potent the inhibitory effect of the sample. Based on the given data, the protease inhibitor cocktail exhibits the most significant inhibitory effect with a negative IC_50_ value (−173.19 mg/mL), suggesting it has a highly potent inhibitory activity against protease enzymes. Among the plant samples, *C. longirostrata* has the lowest IC_50_ value (823.02 mg/mL), indicating that it also possesses a relatively strong inhibitory effect. *L. octovalvis* (910.94 mg/mL) and *C. aconitifolius* (1039.93 mg/mL) have higher IC_50_ values, suggesting they have relatively weaker inhibitory activity compared to the other samples.

### 5.5. FTIR

With the FTIR spectrum, it was found that the most prominent peaks were the same for all three samples, only with different intensities. The most prominent peaks were observed around 650, 1000, and 1650 cm^−1^. This indicates that the composition is similar in all three plants, but the quantity differs. Therefore, a literature search was conducted to identify the major compounds present.

For *C. longirostrata*, the most prominent peak is located at approximately 1650 cm^−1^, followed by a peak at approximately 1600 cm^−1^. According to a study published by Nandiyanto et al. in 2019, this may indicate the presence of an ether and oxy compound, specifically a primary amine NH bend, which typically occurs in the 1650–1590 wavenumber (cm^−1^) range. The second highest peak is observed at 1070 cm^−1^, which, according to the same article, suggests the possibility of an alcohol and hydroxy compound, specifically a primary alcohol C-O stretch, as these compounds typically exhibit a wavenumber of approximately 1050 cm^−1^ [62]. The third most prominent peak, which exhibits a broad shape, reaches its highest point at approximately 640 cm^−1^. According to Coates in 2000, this peak may be attributed to an alkyne C-H bend, as alkyne compounds typically exhibit a wavenumber around 630 cm^−1^ [63]. There is also a broad peak observed between 3000 and 3650 according to the literature, which could correspond to a hydrogen-bonded alcohol OH stretch. This is supported by the wavenumber range of 3550–3200 and the broad nature of the peak [64].

For *C. aconitifolius* and *L. octovalvis*, we observed that the peaks occur in the same locations as *C. longirostrata*, leading us to conclude that they belong to the same functional groups. However, unlike *C. longirostrata*, they also exhibit two weak peaks around 2850–2900, which could be attributed to the presence of C-H stretch alkenes. These alkenes are typically found in the range of 2990–2850 [64].

### 5.6. Statistical Analysis

The primary aim of this analysis was to explore the correlation between the compounds under investigation and enzyme inhibition in plants. The results and interpretation of the dendrogram indicate an initial association between the content of free amino nitrogen and the inhibition of α-Amylase and α-Glucosidase. This suggests that higher levels of free amino nitrogen may lead to greater inhibition of these two enzymes. However, this relationship might not be direct due to the potential involvement of other factors. The observed effects on free amino nitrogen content would likely be secondary effects resulting from changes in nutrient availability or metabolic responses, rather than a direct consequence of enzyme inhibition.

Free amino nitrogen (FAN) refers to nitrogen-containing molecules readily available in the system, such as amino acids, peptides, and ammonium ions [41]. It is primarily derived from protein hydrolysis. The clustering analysis reveals that proteins and FAN are grouped in the same cluster, suggesting a possible association between these two variables; since FAN is predominantly derived from protein hydrolysis this is a reasonable relationship [65]. A more direct relationship may be observed with the content of flavonoids, which are widely occurring polyphenolic compounds [66]. Polyphenols are known to hinder glucose absorption by inhibiting α-Amylase and α-Glucosidase, which are crucial enzymes in carbohydrate digestion [67]. This association can be observed in the first cluster of the dendrogram.

In the second cluster, a relationship between lipase inhibition and fiber content can be observed. Based on the literature, there have only been a limited number of studies investigating the mechanism of how dietary fiber regulates lipid metabolism, with a particular focus on its impact on pancreatic lipase (PL) activity, which is the primary lipolytic enzyme synthesized by the pancreas. One such study conducted by Yu et al. in 2023 revealed that insoluble dietary fiber from citrus peels (CIDF) exhibited both adsorption and inhibitory effects on PL activity [68]. Another study demonstrated that soluble dietary fiber (SDF) also had a noticeable effect on PL activity. Specifically, SDF derived from *K. alvarezii* and *E. denticulatum* showed a reduction in PL activity by 60% and 57%, respectively [69].

In the third cluster, a noticeable relationship can be observed regarding the inhibition of pepsin in connection with the levels of oxalates and FRAP. As we understand it, the FRAP content represents the overall antioxidant activity of electron-donating antioxidants present in a given sample. A higher quantity of antioxidants in a sample corresponds to a greater FRAP content [70]. A study conducted by Salma et al. in 2021 demonstrated that the extract of *E. alte* possesses inhibitory effects on pepsin enzyme, as well as antibacterial and antioxidant activities. However, this alone does not suffice to conclude the existence of a direct relationship between antioxidant content and pepsin inhibition [71]. Our current investigation allows us to potentially confirm this relationship.

Regarding the content of oxalates and enzymatic inhibition, a correlation could plausibly exist, despite the absence of reported evidence in the literature suggesting a direct association. This potential relationship might stem from the involvement of other factors which have yet to be documented.

Based on the information presented earlier and the literature review, this research has the potential to be further developed in the future. Investigating the synergistic mechanisms of herbal ingredients not only has the potential to assist researchers in discovering new phytomedicines or drug combinations but also has the potential to mitigate the risk of negative synergies. Additional clinical research will be essential to validate the reported drug combinations and the elucidated synergistic mechanisms [72]. It is also important to highlight that there are Glycosidase inhibition studies in which it has been found that iminosugars with the gauche–gauche side chain conformations exhibit 6- to 10-fold greater potency compared to isosteric compounds with the gauche–trans conformation. A manno-configured iminosugar with the gauche–gauche conformation is a 27-fold more effective inhibitor than 1-deoxymannojirimycin, which would be worth exploring in future investigations [73].

## 6. Conclusions

The results obtained from the present study suggest the positive therapeutic effects of *L. octovalvis* (Jacq.) P.H.Raven, *C. aconitifolius*, and *C. longirostrata*. The analysis conducted revealed that all three plants exhibited enzymatic inhibition. Upon comparing the plants, it was determined that *C. aconitifolius* had the lowest concentration required for a 50% inhibition in α-Amylase, α-Glucosidase, and lipase, as indicated by the IC_50_ values. In the case of pepsin, *C. longirostrata* demonstrated the lowest IC_50_ value. After analyzing the components of each plant, it can be concluded that there is a correlation between certain bioactive components and enzyme inhibition. Based on our findings, it was determined that certain Polyphenols, particularly flavonoids, possess the ability to impede the activity of α-Amylase and α-Glucosidase, pivotal enzymes involved in the breakdown of carbohydrates. Furthermore, a correlation between lipase inhibition and dietary fiber content was established. Lastly, our study provides support for the presence of a direct association between antioxidant levels and the inhibition of pepsin enzyme.

## Figures and Tables

**Table 1:** Proximal chemical composition of *L. octovalvis*, *C. aconitifolius*, and *C. longirostrata* plant (% dry matter).

Sample	*L. octovalvis*	*C. aconitifolius*	*C. longirostrata*
Moisture (%)	5.53 ± 0.21	4.88 ± 0.34	4.83 ± 0.16
Protein (%)	12.70 ± 1.54	12.52 ± 1.18	14.51 ± 2.04
Lipids (%)	6.67 ± 0.66	5.67 ± 0.50	6.26 ± 0.67
Ash (%)	6.01 ± 0.57	6.66 ± 0.61	0.71 ± 0.14
Carbohydrates (%)	65.30 ± 1.83	70.27 ± 1.02	73.70 ± 2.31
Free sugars (%)	13.99 ± 0.55	15.94 ± 0.80	16.10 ± 0.20
Soluble fiber (%)	28.99 ± 0.75	25.35 ± 1.38	26.34 ± 1.05
Insoluble fiber (%)	36.31 ± 1.13	28.98 ± 1.78	31.25 ± 1.24
Total dietary Fiber (%)	65.30 ± 1.83	54.33 ± 0.42	57.59 ± 2.29

**Table 2:** Bioactive compounds of *L. octovalvis*, *C. aconitifolius*, and *C. longirostrata* plant (% dry matter).

Sample	*L. octovalvis*	*C. aconitifolius*	*C. longirostrata*
Free amino nitrogen (mg/100 g^−1^)	259.67 ± 4.16	225.83 ± 5.55	330.33 ± 12.22
Total phenolics (mg GAE/100 g)	652.96 ± 10.58	740.57 ± 9.43	889.66 ± 2.82
Total flavonoid content (mg of chatequin equivalents/100 g)	337.00 ± 3.61	346.03 ± 4.28	531.53 ± 10.07
DPPH Trolox equivalent (µMTE/g)	2.35 ± 0.06	4.34 ± 0.12	4.52 ± 0.22
Reducing power µg ascorbic acid equivalents	443.36 ± 2.14	572.41 ± 4.93	411.26 ± 3.92

**Table 3:** Antinutrients of *L. octovalvis*, *C. aconitifolius*, and *C. longirostrata* plants (% dry matter).

Sample	*L. octovalvis*	*C. aconitifolius*	*C. longirostrata*
Tannin (mg/g)	14.13 ± 0.14	17.58 ± 0.63	15.34 ± 0.14
Alkaloid (mg/g)	1.12 ± 0.14	1.74 ± 0.11	2.37 ± 0.04
Saponins (mg/g)	2.26 ± 0.30	5.49 ± 0.21	4.42 ± 0.23
Oxalate (mg/g)	4.41 ± 0.22	8.91 ± 0.48	3.32 ± 0.12

**Table 4:** α-Amylase inhibition of *L. octovalvis*, *C. aconitifolius*, and *C. longirostrata* plants (% dry matter).

Sample	*L. octovalvis*	*C. aconitifolius*	*C. longirostrata*	*Acarbose*
100 mg/mL	5.64 ± 0.37	7.18 ± 0.36	8.78 ± 0.48	24.30 ± 0.98
200 mg/mL	22.70 ± 0.47	27.43 ± 1.06	13.60 ± 0.48	36.78 ± 0.42
400 mg/mL	35.77 ± 1.68	44.10 ± 2.19	28.50 ± 0.57	47.79 ± 0.57
600 mg/mL	63.93 ± 0.71	71.89 ± 1.54	47.50 ± 0.80	84.07 ± 0.64
800 mg/mL	86.85 ± 2.25	87.59 ± 1.10	72.53 ± 1.55	95.46 ± 1.15
1000 mg/mL	94.39 ± 0.86	98.37 ± 1.17	85.28 ± 2.76	100.00 ± 0.00
1200 mg/mL	100.00 ± 0.00	100.00 ± 0.00	93.22 ± 0.95	1200.00
IC_50_ mg/mL	519.40	471.39	585.12	338.49

**Table 5:** α-Glucosidase inhibition of *L. octovalvis*, *C. aconitifolius*, and *C. longirostrata* plants (% dry matter).

Sample	*L. octovalvis*	*C. aconitifolius*	*C. longirostrata*	Acarbose
100 mg/mL	5.05 ± 0.48	6.36 ± 0.22	8.36 ± 0.73	19.61 ± 1.45
200 mg/mL	27.71 ± 1.04	32.54 ± 1.42	18.54 ± 0.59	23.92 ± 0.51
400 mg/mL	40.78 ± 2.14	49.21 ± 2.67	33.44 ± 1.04	35.78 ± 0.52
600 mg/mL	68.93 ± 0.15	77.00 ± 1.89	52.44 ± 0.87	55.70 ± 0.70
800 mg/mL	91.86 ± 2.65	92.70 ± 0.63	77.47 ± 1.35	65.55 ± 1.56
1000 mg/mL	99.39 ± 0.96	100.00 ± 0.00	86.54 ± 2.83	74.59 ± 2.29
1200 mg/mL	100.00 ± 0.00	100.00 ± 0.00	93.17 ± 0.97	94.84 ± 0.72
IC_50_ mg/mL	480.50	433.78	579.71	571.61

**Table 6:** Lipase inhibition of *L. octovalvis*, *C. aconitifolius*, and *C. longirostrata* plants (% dry matter).

Sample	*L. octovalvis*	*C. aconitifolius*	*C. longirostrata*	Orlistat
100 mg/mL	4.34 ± 0.29	5.53 ± 0.27	6.76 ± 0.37	35.36 ± 0.09
200 mg/mL	17.48 ± 0.36	21.12 ± 0.81	10.47 ± 0.37	67.13 ± 0.78
400 mg/mL	27.54 ± 1.29	33.96 ± 1.68	21.95 ± 0.44	100.00 ± 0.00
600 mg/mL	49.22 ± 0.55	55.36 ± 1.18	36.58 ± 0.61	-
800 mg/mL	66.87 ± 1.74	67.45 ± 0.84	55.85 ± 1.19	-
1000 mg/mL	72.68 ± 0.66	75.74 ± 0.90	65.67 ± 2.13	-
1200 mg/mL	73.29 ± 0.90	84.27 ± 0.94	71.78 ± 0.73	-
IC_50_ mg/mL	697.17	627.84	677.27	-

**Table 7:** Pepsin inhibition of *L. octovalvis*, *C. aconitifolius*, and *C. longirostrata* plants (% dry matter).

Sample	*L. octovalvis*	*C. aconitifolius*	*C. longirostrata*	Protease Inhibitor Cocktail
100 mg/mL	2.58 ± 0.25	3.32 ± 0.42	4.29 ± 0.21	86.60 ± 2.19
200 mg/mL	7.14 ± 1.29	9.86 ± 1.20	2.92 ± 0.40	100.00 ± 0.00
400 mg/mL	14.79 ± 1.72	19.31 ± 1.35	11.42 ± 0.44	-
600 mg/mL	25.00 ± 1.13	29.46 ± 1.75	17.27 ± 0.09	-
800 mg/mL	39.63 ± 1.85	40.34 ± 1.03	32.76 ± 0.58	-
1000 mg/mL	46.39 ± 0.82	46.88 ± 0.36	46.52 ± 0.33	-
1200 mg/mL	57.85 ± 0.30	56.68 ± 0.59	59.21 ± 0.12	-
IC_50_ mg/mL	910.94	1039.93	823.02	−173.19

**Figure A2:**
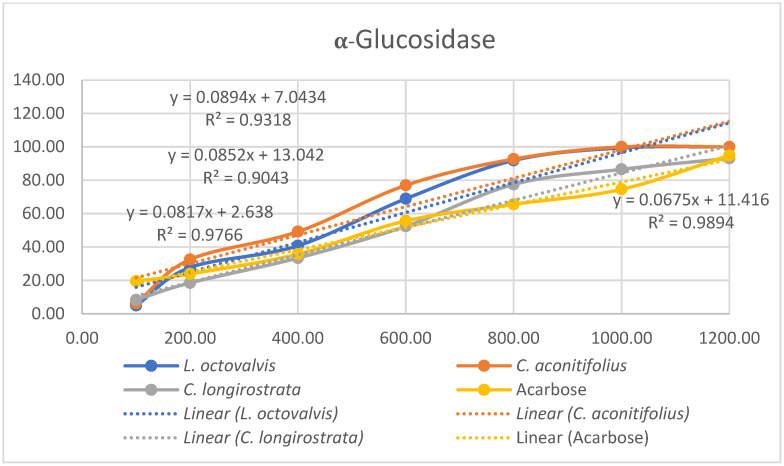
The α-Glucosidase inhibition of *L. octovalvis*, *C. aconitifolius*, and *C. longirostrata* plants expressed as a percentage of dry matter. The data is presented in line charts with trendlines.

**Figure A3:**
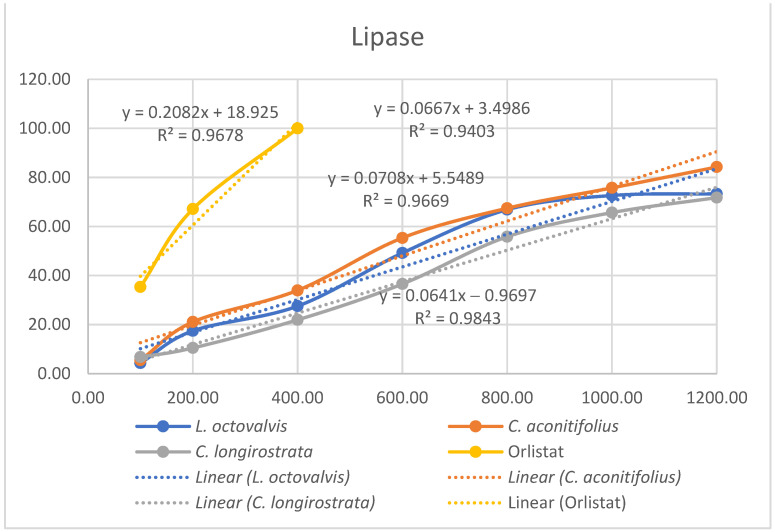
The lipase inhibition of *L. octovalvis*, *C. aconitifolius*, and *C. longirostrata* plants expressed as a percentage of dry matter. The data is presented in line charts with trendlines.

**Figure A4:**
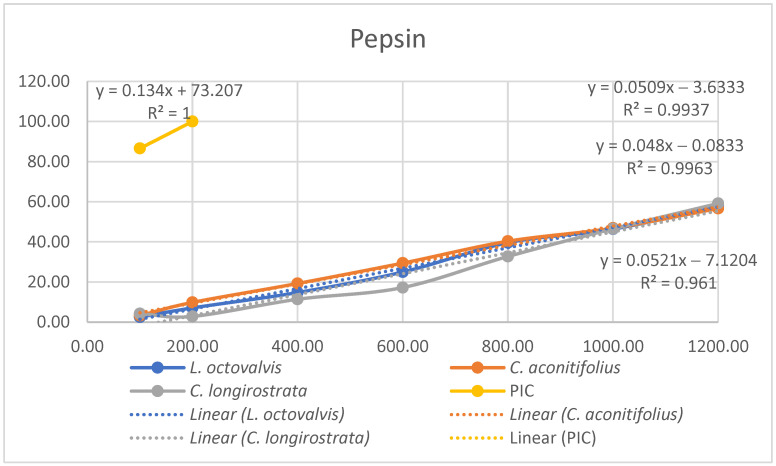
The pepsin inhibition of *L. octovalvis*, *C. aconitifolius*, and *C. longirostrata* plants expressed as a percentage of dry matter. The data is presented in line charts with trendlines.

**Figure A5:**
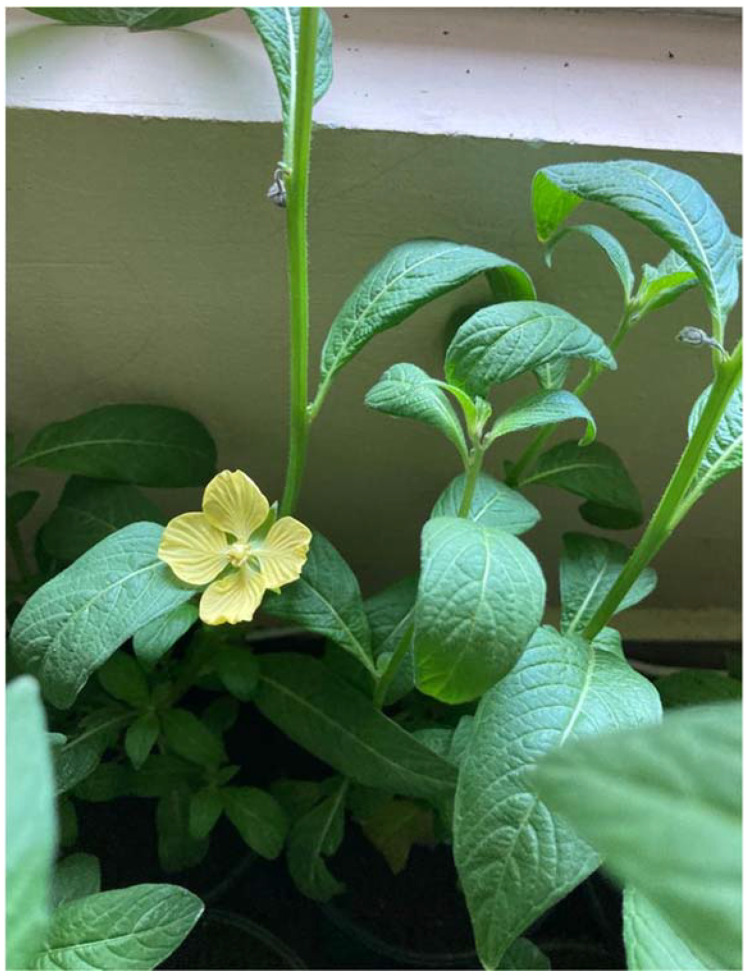
*L. octovalvis* in its reproductive stage, the inflorescence begins to open, and the flowers completely extend.

## Data Availability

The data used to support the findings of this study can be made available by the corresponding author upon request. Additional graphics from enzyme inhibition are shown in the Appendix A.
